# Why Ultrafast Photoinduced CO Desorption Dominates over Oxidation on Ru(0001)

**DOI:** 10.1021/acs.jpclett.2c02327

**Published:** 2022-09-06

**Authors:** Auguste Tetenoire, Christopher Ehlert, J. I. Juaristi, Peter Saalfrank, M. Alducin

**Affiliations:** † Donostia International Physics Center (DIPC), Paseo Manuel de Lardizabal 4, 20018, Donostia-San Sebastián, Spain; ‡ Heidelberg Institute for Theoretical Studies (HITS gGmbH), Schloss-Wolfsbrunnenweg 35, 69118, Heidelberg, Germany; # Interdisciplinary Center for Scientific Computing (IWR), Ruprecht-Karls-Universität Heidelberg, Im Neuenheimer Feld 205, 69120, Heidelberg, Germany; ¶ Departamento de Polímeros y Materiales Avanzados: Física, Química y Tecnología, Facultad de Químicas (UPV/EHU), Apartado 1072, 20080, Donostia-San Sebastián, Spain; § Centro de Física de Materiales CFM/MPC (CSIC-UPV/EHU), Paseo Manuel de Lardizabal 5, 20018, Donostia-San Sebastián, Spain; ∥ Institut für Chemie, Universität Potsdam, Karl-Liebknecht-Straße 24-25, D-14476, Potsdam, Germany

## Abstract

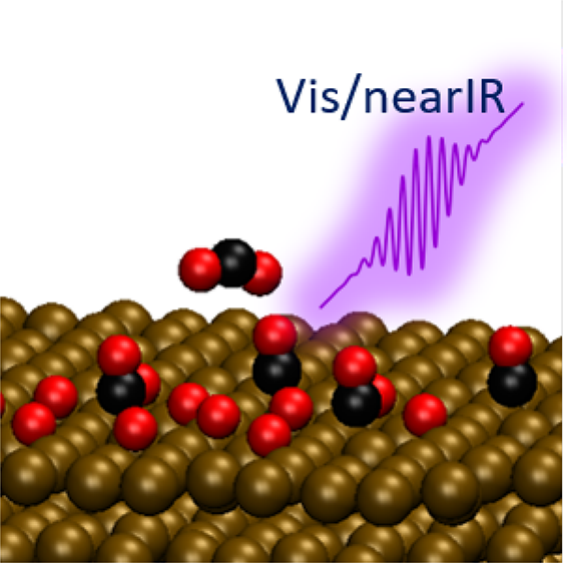

CO oxidation on Ru(0001) is a long-standing example of a reaction that, being thermally forbidden in ultrahigh vacuum, can be activated by femtosecond laser pulses. In spite of its relevance, the precise dynamics of the photoinduced oxidation process as well as the reasons behind the dominant role of the competing CO photodesorption remain unclear. Here we use ab initio molecular dynamics with electronic friction that account for the highly excited and nonequilibrated system created by the laser to investigate both reactions. Our simulations successfully reproduce the main experimental findings: the existence of photoinduced oxidation and desorption, the large desorption to oxidation branching ratio, and the changes in the O K-edge X-ray absorption spectra attributed to the initial stage of the oxidation process. Now, we are able to monitor in detail the ultrafast CO desorption and CO oxidation occurring in the highly excited system and to disentangle what causes the unexpected inertness to the otherwise energetically favored oxidation.

The behavior of Ru(0001) toward CO oxidation is quite unique. By varying the experimental conditions from ambient to low CO pressures, the Ru(0001) surface changes from the most active to the most inert transition metal surface. In particular, early experimental studies carried out for different mixed coverages of O and CO demonstrated that under ultra-high-vacuum (UHV) conditions the oxidation process leading to CO_2_ formation cannot be thermally activated.^1^ Interestingly, the reaction can be initiated by irradiating the system with near-infrared femtosecond laser pulses,^2,3^ while thermally only CO desorption is found.^2^ Photons in this energy range are efficiently absorbed by the metal electrons that can subsequently transfer energy to the adsorbates directly and also indirectly, via the excited surface phonons that result from electron–phonon coupling. The dependence of the reaction probabilities on the delay between two correlated pulses suggests that the oxidation events are initiated thanks to direct coupling of the adsorbed O atoms to the laser-excited electrons, thus explaining that the very same reaction cannot be thermally activated. Still, the competing and indirect laser-induced CO desorption mechanism largely dominates over CO_2_ desorption, with an observed branching ratio of around 35 between CO desorption and CO oxidation.

As remarkable as it is, the precise dynamics of the oxidation process in this emblematic experiment remains unknown. In fact, already the reasons behind the very low probability for CO_2_ formation are unclear because density functional theory (DFT) calculations of the minimum energy path (MEP) of these reactions show that CO_2_ desorption is energetically favored against CO desorption.^3,4^ Altogether, the general questions that naturally arise are how do the ultrafast CO oxidation and CO desorption proceed in the highly excited and nonequilibrated system, and ultimately, what mechanisms make the photoinduced CO desorption more probable than oxidation.

Here we use ab initio molecular dynamics simulations with electronic friction (AIMDEF) that account for the nonequilibrated excited electrons and phonons created in the irradiated (O,CO)/Ru(0001) system to answer these questions. Our simulations allow us to resolve the precise dynamics of the laser-induced desorption and oxidation processes and understand the experimental findings. The large desorption to oxidation branching ratio, which is successfully reproduced here, is caused by the extremely reduced configurational space leading to oxidation as compared to CO desorption. Furthermore, we additionally use our dynamics simulations to calculate the evolution of the O K-edge X-ray absorption spectra (XAS) during the oxidation process. Our real-time spectra reproduce the spectral changes that were observed in ultrafast pump–probe X-ray spectroscopy experiments,^5^ confirming them as fingerprints of the initial stage of the oxidation process. The good agreement achieved here further corroborates the validity of our model to capture and describe the photoinduced oxidation dynamics on the highly excited (O,CO)/Ru(0001) surface.

Photoinduced desorption and oxidation of CO from the (O,CO)-covered Ru(0001) surface is simulated with ab initio classical molecular dynamics using the (*T*_e_, *T*_l_)-AIMDEF methodology^6^ (see also the Supporting Information (SI)^7^). In particular, the laser-excited electrons and concomitant electron-excited phonons are described within the two temperature model (2TM)^8^ as two coupled heat thermal baths, which are characterized by time-dependent electron and lattice temperatures *T*_e_(*t*) and *T*_l_(*t*), respectively. Next, the effect of the excited electrons in the adsorbates dynamics is included by means of Langevin equations of motion, in which electronic friction and random forces model the coupling of the adsorbates to the electronic thermal bath defined by *T*_e_(*t*). Furthermore, effects due to the excited phonons are incorporated by coupling the surface atoms in the upper layers to the Nosé–Hoover thermostat,^9,10^ assuring that the lattice temperature evolves as *T*_l_(*t*). The (*T*_e_, *T*_l_)-AIMDEF method, as well as variants of it, have been widely used to simulate the femtosecond laser-induced dynamics and reactions of adsorbates at metal surfaces.^6,11−20^ The typical short lifetime of the electronic excited states at metal surfaces (of the order of a few femtoseconds) justifies employing a Langevin description because the dynamics evolves in the ground potential energy surface most of the time.^13−15,21^

The experimental (2O+CO)/Ru(0001) honeycomb surface,^5^ in which CO adsorbs atop a Ru atom and the O atoms occupy the second nearest hcp and fcc sites forming a honeycomb arrangement around the CO, is modeled using a periodic slab with five Ru layers and the adlayer (see Figure S1 in SI). The employed (4 × 2) surface cell containing two equivalent adsorbates of each kind is the minimum cell that permits including out of phase movements of the adsorbates and a reliable description of the interadsorbates interactions, that are expected to be relevant at sufficiently large coverages.^6,19,22−26^ All (*T*_e_, *T*_l_)-AIMDEF simulations are performed with vasp(27,28) and the AIMDEF module^19,29−34^ using the same computational parameters and the same van der Waals exchange-correlation functional by Dion et al.^35^ that were used in our previous structural study.^4^ Electronic friction coefficients are calculated with the local density friction approximation (LDFA).^36,37^

The O K-edge XAS have been obtained with the transition potential and Δ-Kohn–Sham methods as implemented in GPAW.^38^ Transition probabilities from the O 1s core orbitals are calculated from a Fermi’s Golden Rule approach using the core and unoccupied orbitals (a Gaussian broadening of 0.5 eV full-width at half-maximum is used). A shifting method beyond the transition potential method is applied to gauge the lowest-energy peak. For the overall spectrum at a single time step, the approach is applied to all individual oxygen atoms, and the final spectrum is obtained by averaging. (For more computational details see SI.^7^)

Table 1 summarizes the results of our simulations for the experimental conditions corresponding to exciting the (2O+CO)/Ru(0001) surface with a 800 nm Gaussian pulse of 110 fs duration and an absorbed fluence *F* = 200 J/m^2^.^2^ The *T*_e_(*t*) and *T*_l_(*t*) curves calculated with the 2TM for these experimental conditions are shown in Figure 1 (input parameters as in refs (13), (18), and (19)). We run 200 (*T*_e_, *T*_l_)-AIMDEF trajectories with a total simulation time of 4 ps each,^39^ the system being initially thermalized at 100 K. The desorption (oxidation) probability per molecule is obtained by dividing the total number of CO (CO_2_) desorbing molecules^40^ by the total number of trajectories and the total number of CO in the cell. The result of our (*T*_e_, *T*_l_)-AIMDEF simulations is clear. As observed in experiments, after photoexcitation both CO desorption and CO_2_ formation take place, and the former largely dominates over the latter. The corresponding calculated probabilities of 18.25% and 0.5% yield a branching ratio between the two processes in nearly perfect agreement with the experimental values *P*_des_(CO)/*P*_des_(CO_2_) = 35^2^ and 31.^3^ The agreement must be considered as qualitative because of the limited statistics we have for the oxidation process (two oxidation events in 200 trajectories) and our integration time of 4 ps (note that some oxidation and desorption events may occur beyond this interval, as found for CO desorption from Pd(111)^26^), but it relates already the validity of our nonequilibrium two-temperature picture. As shown below, the fact that the XAS features attributed to the oxidation reaction path are also well reproduced makes our simulations all the more reliable.

**Figure 1:**
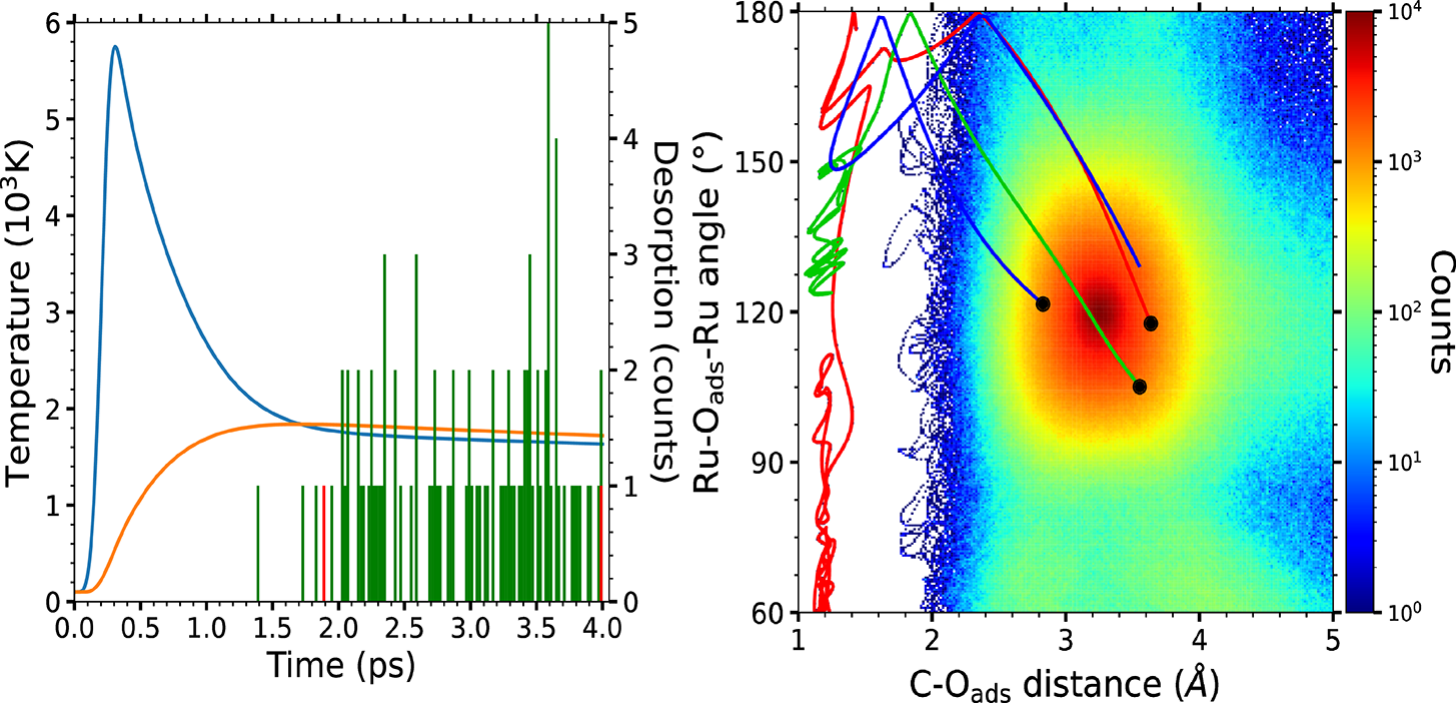
Left: Electronic (blue curve) and phononic (orange curve) temperatures (left *y*-axis) used in the simulations. The peak of the pump laser pulse is at 236 fs. The instants at which the desorption and oxidation events occur are respectively plotted by green and red histograms (right *y*-axis), using 20 fs as bin width. Right: Density plot of the instantaneous in-plane dihedral angle Ru–O_ads_–Ru and the distance between that O_ads_ and the C atom in its nearest CO (see SI(7) for calculation details). For clarity, the values of the two oxidation events are shown by a red and a green line, respectively. The blue line corresponds to the third O adsorbate that reaches the transition state, but it cannot recombine. Only the interval of 50 fs before and after reaching the transition state for oxidation is shown by each line. The black circles correspond to the starting points of these three lines.

**Table 1:** AIMDEF Desorption and Oxidation Probabilities, *P*_des_(CO) and *P*_des_(CO_2_), for (2O+CO)/Ru(0001) and *F* = 200 J/m^2^. The CO to CO_2_ Branching Ratio Is Compared to the Available Experimental Values^2,3^

simulation	*P*_des_ (CO)	*P*_des_ (CO_2_)	ratio
(*T*_*e*_, *T*_l_)-AIMDEF	18.25%	0.5%	36.5
refs (2) and ^3^	-	-	35, 31

The results of the (*T*_e_, *T*_l_)-AIMDEF simulations provide us with a detailed understanding of the laser-induced oxidation process. The fact that CO desorption dominates over CO oxidation is particularly striking considering that previous DFT calculations of the minimum energy reaction paths showed that desorption requires around 0.38 eV of additional energy compared to oxidation at this coverage.^4^ The natural question that arises is whether the laser-induced oxidation dynamics does or does not proceed through that minimum energy oxidation path, in which the less bound O_fcc_ abandons its adsorption well, crosses the bridge site between two Ru atoms, and recombines with the nearby CO that tilts to form the chemisorbed bent CO_2_ (hereafter denoted bCO_2_). The selected snapshots depicted in Figure 2 for one of the trajectories confirm that this is the case. In both trajectories leading to CO_2_ desorption, it is an O_fcc_ adsorbate that recombines with CO. During the first picosecond upon arrival of the laser pulse, all the adsorbates become highly vibrationally excited. Thus, the CO molecules, although bound to the Ru atom below, tilt profoundly, while the O_fcc_ and O_hcp_ adsorbates explore the upper part of the wells, approaching the bridge site regions. It is in the interval *t* ≃1580–1600 fs that the recombining O_fcc_ crosses the bridge site and the chemisorbed bCO_2_ is formed at *t* ≃1600–1630 fs. From the chemisorbed state the molecule evolves toward the physisorbed linear CO_2_ (*t* ≃1820 fs) and finally desorbs at *t* ≳ 2 ps. Altogether, the figure details the complexity of the oxidation process as compared to the simpler CO desorption dynamics, which does not involve intermediate states and barriers.^4^ By analyzing the details of the dynamics, we conclude that it is the reduced configurational space of the former, and particularly the access to the transition state, that explains why the energetically less favorable CO desorption process dominates. This is inferred from the density plot of Figure 1 showing the distribution of the instantaneous Ru–O_fcc, hcp_–Ru (in-plane) dihedral angles and the distance between that adsorbed O and the C atoms in the two nearest CO (see SI(7)). A successful recombination requires that the adsorbed O reaches the bridge site (characterized by a dihedral angle of 180°) and encounters a CO slightly displaced toward the ahead hollow site and adequately tilted to facilitate the C–O bonds (C–O_fcc, hcp_ distances smaller than 2 Å). These are the properties defining the transition state. As shown in the figure, the access to the bridge site is rather probable, but only on three of these occurrences does the excited O find a CO correctly oriented for recombination. The fact that only two of the three trajectories shown in the figure end as desorbing CO_2_ shows that the existence of recrossing events in the transition state makes the oxidation process even more difficult. It is also remarkable to observe that the full oxidation process, which unavoidably involves various intermediate states, lasts a few picoseconds. This is explicitly shown in Figure 1, where the instants for desorption and oxidation are plotted together with the time evolution of the electronic and lattice temperatures.^41^ In summary, not only the energetics determines reactivity, but the dynamics plays an important role, too.

**Figure 2:**
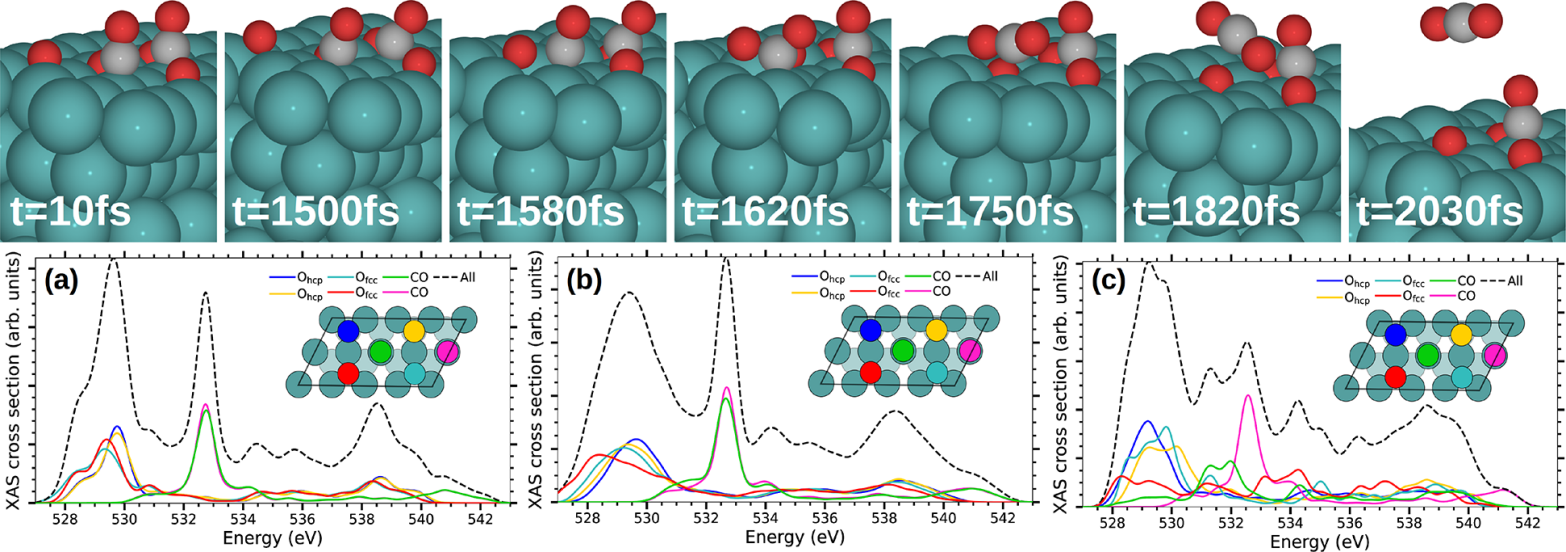
Top: Snapshots of a representative CO oxidation dynamics obtained in the (*T*_e_, *T*_l_)-AIMDEF simulations (blue, red, and gray spheres correspond to Ru, O, and C atoms, respectively). The AIMDEF simulation time is indicated in each panel (*t* = 0 as in Figure 1). Bottom: Time averaged O–K XAS cross section calculated at characteristic time intervals during the oxidative desorption process in the selected trajectory: (a) initial strong excitation of the adsorbates (0–1250 fs), (b) access to the transition state ruling the reaction, in which the recombining O_fcc_ reaches the bridge site that separates it from the nearest CO (1250–1580 fs), and (c) formation of the chemisorbed bent CO_2_ (1600–1630 fs). Each color curve shows the contribution of the corresponding colored O atom depicted in the surface unit cell plotted as an inset. Black dashed curves show the total time-averaged XAS.

Our dynamics simulations confirm the interpretation provided in ref (5) on the changes observed in the time-resolved XAS experiments. However, that interpretation was based on static DFT calculations under the assumption, questionable under nonequilibrium conditions, that the oxidation process follows the minimum energy reaction path with the surface at equilibrium. This approach fully neglects that the reaction proceeds in an extremely dynamically perturbed system. Our dynamics simulations are free of those assumptions and treat explicitly the highly excited environment. As a consequence, they provide an explanation of the measured time-dependent XAS and permit us to unravel the mechanisms that govern them. The calculated O K-edge XAS is shown in Figure 2a–c at selected time intervals characterizing the initial stages in the oxidation dynamics followed by the trajectory shown in the same figure. In agreement with experiments, the absorption peak at ∼530 eV associated with the recombining O_fcc_ shifts and broadens toward lower energies as the adsorbate approaches the Ru–Ru bridge site [compare panels (a) and (b)]. Few tens of femtoseconds later, the absorption peak assigned to the CO 2π* excitation (∼533 eV) red shifts as the distance between the recombining O_fcc_ and CO decreases, and they start to form the chemisorbed bCO_2_ [compare panel (c) to (a) and (b)]. Also in agreement to experiments, at this oxidation stage we observe that the initial wide weak peak at ∼539 eV, which is equally contributed by the four O adsorbates [panel (a)], is transformed into a broad structure that extends from about 536 to 540 eV. Comparison of panels (a) and (b) to (c) shows that the change is caused by the reacting O_fcc_ (compare the red curves).

The time-resolved O K-edge XAS spectra plotted in Figure 3 for each adsorbate in the simulation cell allow us to track in detail the contribution of each adsorbate to the changes observed in the spectra associated to the trajectory of Figure 2. During the first initial femtosecond, the dominant absorption peaks of O_hcp_ and O_fcc_ are slightly shifted from each other because of their minor different chemical environments. In all cases, the main absorption peak evolves during the first hundreds of fs following a zigzag structure that reflects the small displacements experienced by the adsorbates as a consequence of the electronic and phononic excitations. The observed red and blue shifts in both O_hcp_ and O_fcc_ correlate with the instants at which adsorbates approach and separate from the nearby Ru atoms. During the interval lasting from about 1.25 to 1.6 ps, the recombining O_fcc_ approaches and successfully reaches the bridge position that separates it from the adsorbed CO. This stage is identified in its corresponding spectra by a profound net red shift of the dominant absorption peak from ∼530 eV to ∼528 eV that is not observed in any of the other O adsorbates. The comparatively small changes (oscillations) in the two CO spectra underlines that the molecules remain firmly bound atop the Ru atom, except for the minor displacements associated to frustrated rotations and translations. At 1.6 ps the absorption peak of the recombining CO vanishes, while a new broaden and less intense peak appears in the range 531–532 eV. This new structure is ascribed to the starting point in the formation of the chemisorbed bCO_2_. The additional analysis of the partial density of states and partial electron densities allows us to confirm the formation of all the bCO_2_ orbitals at 1.63 ps (see Figure S4 in SI). The nascent molecule stays about 100 fs in this state before definitely breaking the C–Ru bond and reaching the physisorbed linear CO_2_. Although experimentally inaccessible, note that the absorption spectra in the physisorption state are basically identical for the O atoms, as expected.

**Figure 3:**
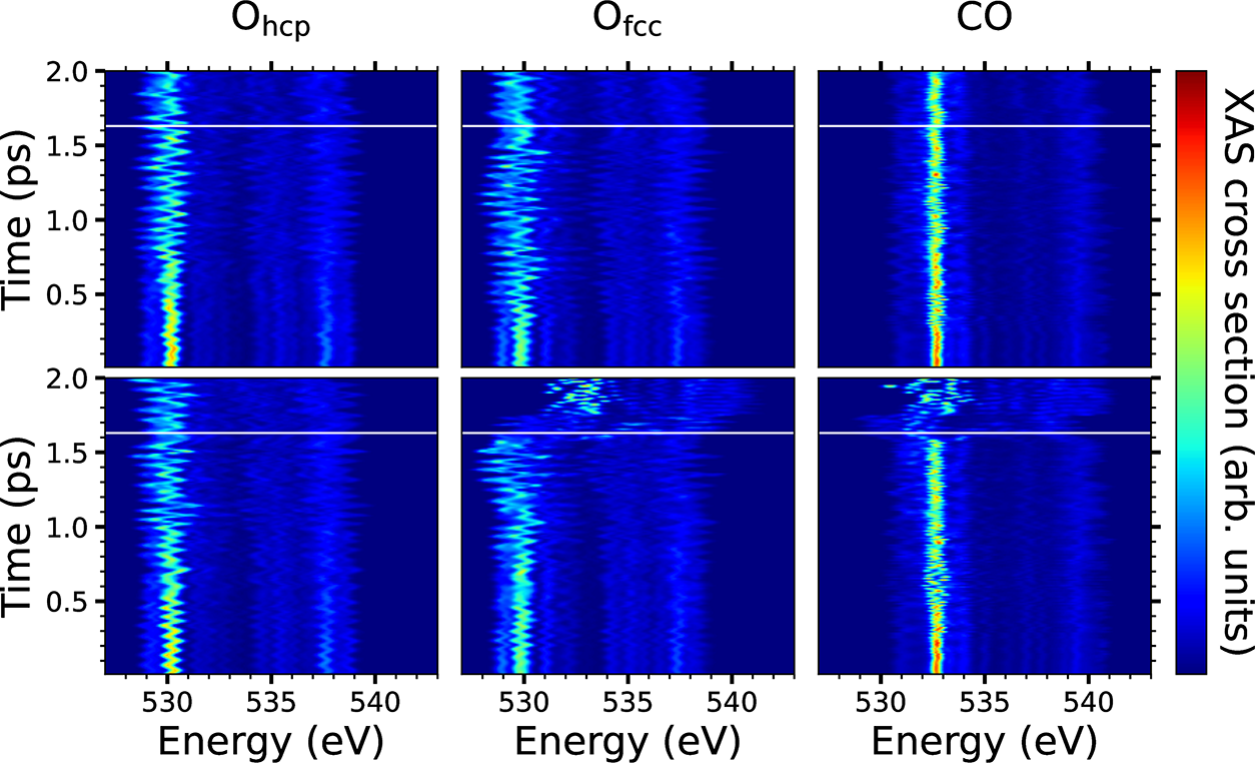
Instantaneous O–K XAS of the trajectory shown in Figure 2, resolved for each O atom in the simulation cell: O_hcp_ (left panels), O_fcc_ (middle panels), and CO (right panels), with the recombining O_fcc_ and CO being at the bottom. For illustrative purposes, the first instant at which the O_fcc_–C distance matches the bCO_2_ internuclear distance (1.14 Å) is shown by a white line in each plot.

In conclusion, our ab initio molecular dynamics simulations that describe the laser-excited system in terms of nonequilibrated time-dependent electronic and phononic temperatures have successfully reproduced the main features observed in femtosecond laser experiments performed on Ru(0001) covered with a mixed adlayer of O and CO: (i) the photodesorption of both CO and CO_2_, (ii) the large branching ratio between desorption and oxidation that exceeds 1 order of magnitude, and (iii) the changes in the O K-edge X-ray absorption spectra that were associated with the initial stage of the oxidation. Because of these simulations we can monitor in detail the elementary steps of the desorption and the oxidation dynamics promoted by the laser and determine the reaction paths in the excited system that explain why CO desorption dominates over the energetically favored oxidation. It is the O adsorbed at the fcc sites that primarily recombines with the adsorbed CO, following basically the intermediate extreme states of the minimum energy oxidation path. The reason behind the unexpected inertness to the otherwise energetically favored oxidation is 2-fold: (i) the difficult access to the transition state region, that requires the O atom crossing the bridge site and finding the CO conveniently close and tilted to form the chemisorbed bent CO_2_ and (ii) the fact that this access does not guarantee a successful recombination.

## Supporting Information Available

Further information on the (*T*_*e*_, *T*_*l*_)-AIMDEF and XAS simulations (PDF)
