# Supplementary material for: Why Ultrafast Photoinduced CO Desorption Dominates over Oxidation on Ru(0001)
Source: J Phys Chem Lett. 2022 Sep 6;13(36):8516–21. doi: 10.1021/acs.jpclett.2c02327 (PMC9486938; doi:10.1021/acs.jpclett.2c02327)
Supplement: Supplementary file 1 — jz2c02327_si_001.pdf [file jz2c02327_si_001.pdf]

Supporting Information: Why Ultrafast Photo-induced CO Desorption Dominates over Oxidation on Ru(0001)

Auguste TETENOIRE,[*,†] Christopher Ehlert,[*,‡] J. I. Juaristi,[*,¶,§,†] Peter Saalfrank,[*,‖] and M. Alducin[*,§,†]

† *Donostia International Physics Center (DIPC), Paseo Manuel de Lardizabal 4, 20018 Donostia-San Sebastián, Spain*

‡ *Heidelberg Institute for Theoretical Studies (HITS gGmbH), Schloss-Wolfsbrunnenweg 35, 69118 Heidelberg (Germany), and Interdisciplinary Center for Scientific Computing (IWR), Ruprecht-Karls-Universität Heidelberg, Im Neuenheimer Feld 205, 69120 Heidelberg (Germany)*

¶*Departamento de Polímeros y Materiales Avanzados: Física, Química y Tecnología, Facultad de Químicas (UPV/EHU), Apartado 1072, 20080 Donostia-San Sebastián, Spain*

§ *Centro de Física de Materiales CFM/MPC (CSIC-UPV/EHU), Paseo Manuel de Lardizabal 5, 20018 Donostia-San Sebastián, Spain*

‖ *Institut für Chemie, Universität Potsdam, Karl-Liebknecht-Straße 24-25, D-14476 Potsdam, Germany*

E-mail: auguste.tetenoire@dipc.org; christopher.ehlert@h-its.org; josebainaki.juaristi@ehu.eus; peter.saalfrank@uni-potsdam.de; maite.alducin@ehu.eus

Dynamics simulations

Theoretical model

The $(T_\mathrm{e}, T_\mathrm{l})$-AIMDEF methodology is described and justified in detail in ref. S1. Here, we will quickly review the equations that are used. As a first step, the response of the metal surface to the near-infrared laser pulse is described within the two-temperature model (2TM),[S2] in which the laser-induced electronic and concomitant (electron-induced) phononic excitations are represented by two coupled hot thermal baths that are respectively characterized by two distinct time-dependent temperatures $T_\mathrm{e}(t)$ and $T_\mathrm{l}(t)$. These temperatures are obtained by solving the two following equations:

$$\begin{aligned} C_e \frac{\partial T_e}{\partial t} &= \frac{\partial}{\partial z} \kappa \frac{\partial T_e}{\partial z} - g\left(T_e - T_l\right) + S(z,t)\,, \\ C_l \frac{\partial T_l}{\partial t} &= g(T_e - T_l)\,, \end{aligned} \tag{S1}$$

where C_e is the the electron heat capacity, C_l is the phonon heat capacity, κ is the electron thermal conductivity, g is the electron-phonon coupling constant, z the perpendicular position relative to the surface and $S(z,t)$ is the absorbed laser power per unit volume that depends on the shape, wavelength, and fluence of the applied pulse.

Once $T_e(t)$ and $T_l(t)$ are known, the classical equations of motion of the adsorbates and surface atoms are solved ab initio using a modified version of the VASP code, in which we implemented the $(T_\mathrm{e}, T_\mathrm{l})$-AIMDEF methodology as follows. The effect of the laser-excited electrons on each adsorbate is described through the following Langevin equation:

$$m_i \frac{d^2\mathbf{r}_i}{dt^2} = -\nabla_{\mathbf{r}_i} V(\mathbf{r}_1, ..., \mathbf{r}_N) - \eta_{e,i}(\mathbf{r}_i)\frac{d\mathbf{r}_i}{dt} + \mathbf{R}_{e,i}[T_e(t), \eta_{e,i}(\mathbf{r}_i)]\,, \tag{S2}$$

where m_i, $\mathbf{r}_i$, and $\eta_{e,i}$ are the mass, position vector, and electronic friction coefficient of the ith atom conforming the set of adsorbates. The first term in the right hand side of the equation is the adiabatic force that depends on the position of all (adsorbates and surface)

atoms. The second and third terms are the electronic friction and electronic stochastic forces, respectively, mimicking the effect of the electronic excitations and deexcitations on the adsorbates. Both forces are related through the fluctuation-dissipation theorem, being $\mathbf{R}_{e,i}$ modeled by a Gaussian white noise with variance

$$\mathrm{Var}[\mathbf{R}_{e,i}(T_e, \eta_{e,i})] = \frac{2k_B T_e(t)\eta_{e,i}(\mathbf{r}_i)}{\Delta t}\,, \tag{S3}$$

where k_B and Δt are the Boltzmann constant and the time-integration step, respectively.

The electronic friction coefficient for each adsorbate atom $\eta_{e,i}$ is calculated within the local density friction approximation (LDFA).[S3,S4] Therefore, $\eta_{e,i}$ depends on the value of the bare surface electronic density at the position of the atom i forming the adsorbate $n_{\mathrm{sur}}(\mathbf{r}_i)$ and it is calculated as the friction coefficient of the atom moving in a homogeneous free electron gas of the same density. This calculation is performed to all orders in the nuclear charge in terms of the DFT potential of an impurity embedded in jellium.[S5,S6] As proposed in,[S7,S8] the electron density of the bare surface $n_{\mathrm{sur}}(\mathbf{r}_i)$ is calculated at each integration step in our simulations by subtracting the contributions of the adsorbates (calculated with the Hirshfeld partitioning scheme[S9]) from the self-consistent electronic density.

The equations of motion of the surface atoms account for the heating of the surface lattice due to the electronic excitation generated by the laser pulse. In our case, this is done by coupling the Ru atoms of the first two surface layers to a thermal bath described by a Nosé-Hoover thermostat,[S10,S11] in which the temperature is the time dependent temperature $T_l(t)$. Thus, the equations of motion of these surface atoms j with mass m_j and position vector $\mathbf{r}_j$ are the following:

$$m_j \frac{d^2\mathbf{r}_j}{dt^2} = -\nabla_j V(\mathbf{r}_1, ..., \mathbf{r}_N) - m_j\, \xi\, \frac{d\mathbf{r}_j}{dt}\,, \tag{S4}$$

$$\frac{d\xi}{dt} = \frac{1}{Q}\left(\sum_j m_j \left|\frac{d\mathbf{r}_j}{dt}\right|^2 - 3Nk_B T_l\right), \tag{S5}$$

where N is the number of atoms of the first two layers in our simulation cell, Q is a parameter with dimensions of energy×time^2 that acts as the mass of the dynamical variable s and $\xi = Q^{-1}sp_s$ is the thermodynamic friction coefficient, being p_s the conjugated momentum associated with s.[S12]

Finally, the movement of the third layer atoms k is described by the classical Newton equations of motions and the adiabatic approximation:

$$m_k \frac{d^2\mathbf{r}_k}{dt^2} = -\nabla_k V(\mathbf{r}_1, ..., \mathbf{r}_N)\,, \qquad \text{(S6)}$$

where m_k and $\mathbf{r}_k$ are its corresponding mass and position vector. Finally, the fourth and fifth Ru layers are kept frozen in our simulations.

General DFT computational settings

All (T_e,T_l)-AIMDEF simulations are performed with VASP[S13,S14] (version 5.4) and the AIMDEF module[S7,S8,S15–S19] using the supercell depicted in Figure S1. This periodic supercell is defined by a (4×2) surface unit cell and a vector length along the surface normal of 30.2225 Å. Within this supercell, the (0.5ML O+0.25ML CO)/Ru(0001) surface is described by five layers of Ru atoms and one (2O+CO) layer adsorbed on the topmost Ru surface layer. The Ru topmost layer is separated from the bottom of the periodic Ru slab by about 19 Å of vacuum. As shown in the figure, each CO adsorbs atop a Ru atom, while the O atoms occupy the second nearest hcp and fcc sites forming a honeycomb arrangement around the CO.

During the AIMDEF simulations, the adiabatic forces are calculated with non spin-polarized DFT using the van der Waals exchange-correlation functional proposed by Dion *et al.*[S20] and the same computational parameters that were used in our previous structural study on CO desorption and oxidation at different coverages.[S21] Specifically, the electronic ground state is determined by minimizing the system total energy up to a precision of 10^{-6} eV.

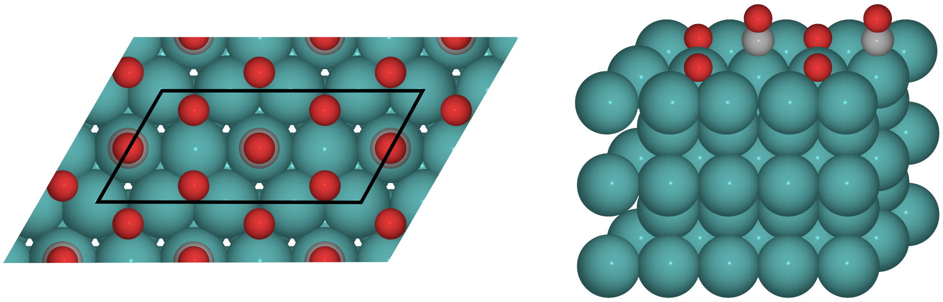

Figure S1: Top (left) and perspective (right) views of the energetically favored honeycomb structure obtained for the 0.5 ML O + 0.25 ML CO coverage on Ru(0001). The black parallelogram depicts the (4×2) surface unit cell used in all the AIMDEF calculations. Color code: O atoms in red, C in gray, and Ru in blue. Note that within the surface unit cell (top view), the atop CO adsorbates correspond to the middle row of adsorbates, while the O_{fcc} and O_{hcp} adsorbates are in the bottom and top rows, respectively. For clarity, the periodic images of the O and CO adsorbates in the perspective view are not shown.

Integration in the Brillouin zone is performed using a Γ-centered 3×6×1 Monkhorst-Pack grid of special **k** points[S22] and the Methfessel and Paxton scheme of first order with a broadening of 0.1 eV to describe partial occupancies of each state.[S23] The latter are expanded in a plane-wave basis set with an energy cut-off of 400 eV, whereas the electron-core interaction is treated with the projected augmented wave (PAW) method[S24] that is implemented in VASP.[S25]

Initial conditions

Starting with the relaxed (2O+CO)/Ru(0001) surface, we run a preliminary simulation in which the three outmost Ru layers are equilibrated at 100 K during 20 ps using the Nosé-Hoover thermostat[S10,S11] [Eqs. (S4) and (S5)], while the (2O+CO) adlayer follows an adiabatic dynamics [Eq.(S6)]. The adlayer, being in contact to the thermalized Ru(0001) surface, becomes progressively thermalized at the end of this simulation. The initial positions and velocities of the moving atoms (i.e., the adlayer and the three topmost Ru layers) are randomly taken from the data generated in the last 5 ps of the thermalization trajectory. We have verified that the selected 200 trajectories reasonably reproduced the expected Maxwell-Boltzmann distribution.

All dynamics simulations are performed with the Beeman integrator implemented in our AIMDEF module[S15] using a time step of 1 fs.

Density plot calculation

The density plot showing the distribution of the instantaneous Ru-$O_{fcc,hcp}$-Ru (in-plane) dihedral angle and the distance from that adsorbed O to the C atom in the nearest CO (right panel of Figure 1 in the main text) is calculated as follows.

First, for each instantaneous configuration extracted from the data set formed by the 200 (T_e,T_l)-AIMDEF trajectories and each integration step (4000 points per trajectory), we calculate for each O adsorbate (O_{ads}) in the unit cell the three Ru-O_{ads}-Ru (in-plane) dihedral angles Θ that the adsorbate forms with the three (initially) nearest Ru atoms in the topmost layer (i.e., the three Ru atoms that were the nearest neighbors to the corresponding O_{ads} in the initial configuration), see Figure S2.

Next, for the same instantaneous configuration and the same O_{ads}, we calculate the minimum distances $d_{\text{O-C1}}$ and $d_{\text{O-C2}}$ from O_{ads} to any of the images of the two nonequivalent C atoms in our unit cell (labeled C1 and C2 in Figure S2). In practice, we generate eight periodic images of the C1 and C2 atoms in the unit cell by applying $\pm\vec{a}$ and/or $\pm\vec{b}$ translations. Subsequently, we calculate the distances from O_{ads} to C1 and C2 in the unit cell and to their 8 images in adjacent cells, and the minimum distance for each nonequivalent atom is selected. This process is repeated for each of the four nonequivalent O_{ads} in the unit cell. Note that for each instantaneous configuration and each O_{ads} we extract six pairs of points (Θ,$d_{\text{O-C}}$) corresponding to three dihedral angles and two distances ($d_{\text{O-C1}}$ and $d_{\text{O-C2}}$). The distribution of all (Θ,$d_{\text{O-C}}$) points is represented as the density plot shown in the right panel of Figure 1 in the main text, being the angle and distance bin widths 0.4° and 0.02 Å, respectively.

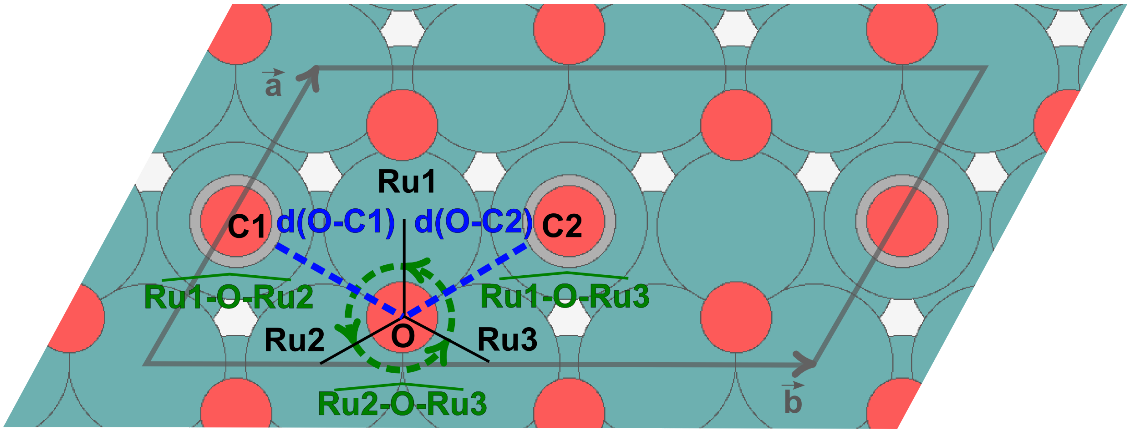

Figure S2: Scheme showing, as an example, the three Ru-O_{ads}-Ru in-plane dihedral angles (in green) and the two O_{ads}-C distances (in blue) calculated for one of the four O adsorbates in the unit cell.

Additional information on the dynamics simulations results

Figure S3 shows, as density plots, the distribution of the center of mass positions over the Ru(0001) surface that are visited by each adsorbate along the 200 (T_e,T_l)-AIMDEF trajectories. Initially, all adsorbates are localized at or nearby their corresponding adsorption sites (note that in the (T_e,T_l)-AIMDEF simulations the adsorbates have previously been thermalized to $\simeq$100 K and, therefore, are slightly displaced from the equilibrium position). This situation is identified in the figure by the highest density areas depicted in red. As time evolves, the adsorbates move progressively away from their initial position, what we identify by the areas changing from orange, yellow, green and blue in the density plots.

As written in the main text, we have additionally calculated the projected density of states (PDOS) at certain instants along the trajectory shown in Figure 2 to follow in more detail the formation of the chemisorbed bent CO_2 (bCO_2). At 1600 fs, we start to observe hybridization of the more energetic O_{fcc} and CO occupied states, but it is at 1630 fs when we identify all the bCO_2 orbitals. This is shown in Figure S4. Note that in the VASP implementation, the PDOS is obtained by projecting the Kohn-Sham wave function $\phi_{n\mathbf{k}}$ onto spherical harmonics Y^i_{lm} centered at each ion position i. The PDOS shown in the figure corresponds to the sum of the s and p projections centered at the atoms involved in the CO_2

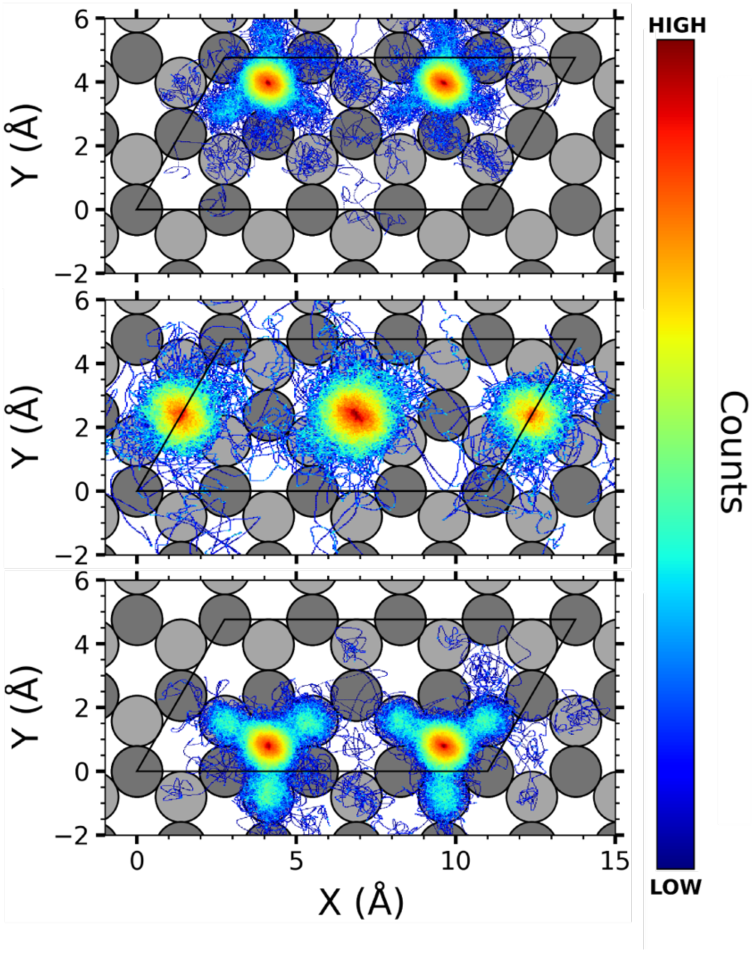

Figure S3: Density plots of the (x, y) positions over the surface for O_{hcp} (top panels), CO center of mass (middle panels), and O_{fcc} (bottom panels). Only the positions of the adsorbates that remain adsorbed on the surface are shown. Note that the initial positions of the atoms in the supercell are taken from a preliminary AIMD simulation in which the system is thermalized at 100 K. As a consequence, the AIMDEF trajectories do not start with the adsorbates located exactly on their equilibrium position, but slightly displaced by $\pm\Delta\mathbf{r}$. The latter means that, in the middle panel, the initial position of the CO adsorbed at the edge of the cell is in some trajectories on the left edge and in others on the right edge of the cell, but both density plots correspond to the same CO.

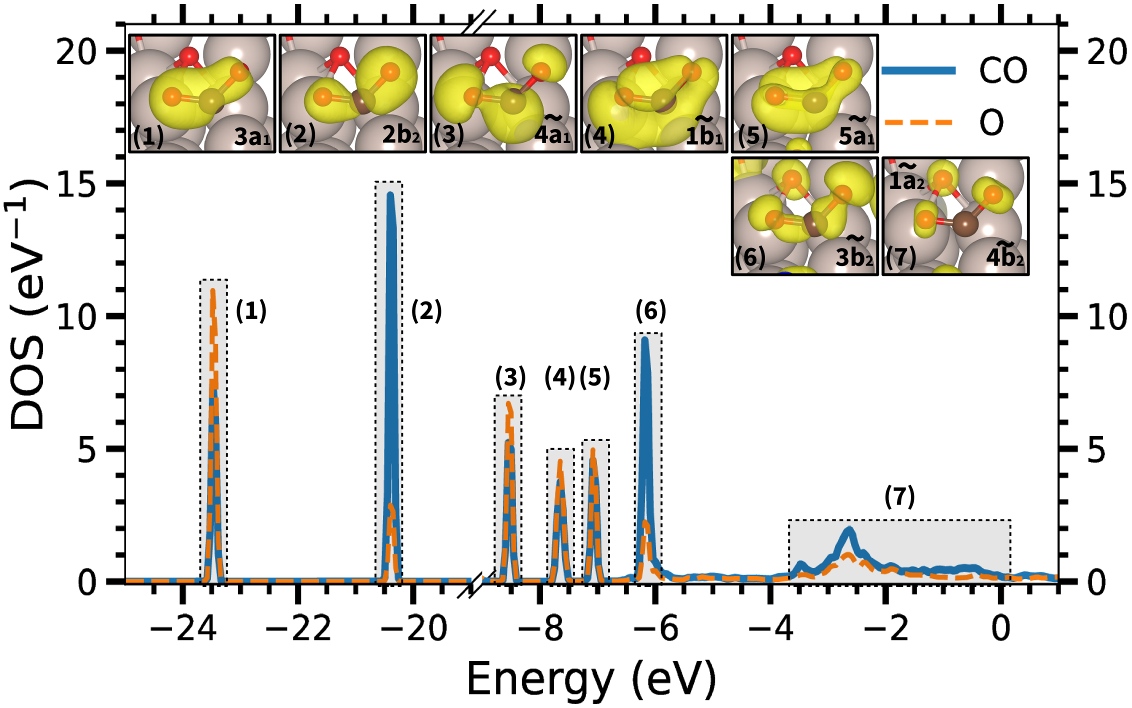

Figure S4: Projected density of states and partial electron densities showing that at t=1630 fs the chemisorbed bCO_2 is completely formed in the trajectory depicted in Figure 2 of the main text. In the PDOS plot, the sum of the s- and p-like projections centered at the atoms forming the recombining CO is depicted by the blue curve, while the dashed orange curve shows the sum of the s and p projections centered at the recombining O_{fcc} atom. The insets (1) to (7) show the partial electron densities (PED) calculated for each of the (1) to (7) PDOS peaks identified in the main plot. (The energy interval used in each PED calculation is marked by a gray rectangle in the PDOS plot.) The bCO_2 orbital associated to each PED is written in the inset (when applicable, the tilde remarks that the orbital hybridizes with the Ru(0001) states[S26]). Note that the PED associated to peaks (6) and (7) also include the contribution in this energy range of the adsorbed O that should not be confused with the bCO_2 orbitals.

formation. For each of the PDOS peaks we have additionally calculated the corresponding partial electron densities to facilitate the identification of the bCO_2 orbitals (we followed the energy diagram of ref. S26 showing how the linear CO_2 states change as the molecule bends and ref. S27 showing some of the bCO_2 orbitals).

Simulation of X-ray absorption spectra

For O K-edge X-ray absorption spectra, a transition state potential method in combination with a Delta-Kohn-Sham method has been applied. All calculations are performed with the GPAW program,[S28] a real-space grid density functional theory program that uses the projector augmented wave (PAW) approach.[S29] For each calculation, the initial unit cell was duplicated along the shortest axis, mainly to avoid core-hole/core-hole interactions and the three lowest Ru-layers have been removed from the snapshots. Further, a grid spacing of 0.2Å and the PBE (Perdew-Burke-Ernzerhof)[S30,S31] exchange-correlation functional have been used. Choosing PBE offers a slight computational performance advantage compared to a non-local van der Waals functional, while the choice of the exchange-correlation functional mainly affects the absolute position of the overall spectrum (we expect by less than 5 eV for the O K-edge). The shape and relative positions of the resonances are less affected (see, for example[S32]). All calculations are done at the Γ-point and in a spin polarized fashion to account for an unequal number of spin-up and spin-down electrons during the simulation procedure (see below). To obtain the total absorption cross section, $\sigma_T(\omega, t_n)$, at a single timestep t_n, the following calculations have been done:

1. Ground state calculation, to obtain the electronic ground state energy, E_{gs}.

2. For each excitation center i (unique oxygen atoms in the system), a transition potential calculation is performed. This method utilizes an artificial auxiliary state, where the core-orbital (here oxygen 1s) at a single excitation center is occupied by half an electron. The core orbital occupation number is set by using a pre-generated PAW potential.

After the transition potential state is found variationally, the contribution of the i-th excitation center is given by a Fermi's Golden Rule expression:

$$\sigma^i(\omega, t_n) = C\omega \sum_f \sum_{s=x,y,z} |\langle \psi_i \mid s \mid \psi_f \rangle|^2 \delta(\epsilon_f - \epsilon_i - \hbar\omega) \quad , \tag{S7}$$

where C is a constant, $\hbar\omega$ the excitation energy, ψ_i is the initial state (O 1s orbital) at the excitation center "i". ψ_f represents all possible final states (unoccupied bands). ϵ_i and ϵ_f correspond to the orbital (band) energies. $\langle \psi_i \mid s \mid \psi_f \rangle$ are transition dipole matrix elements ($s = x, y, z$). To account for finite experimental resolution, the δ-functions are replaced by Gaussians with a full-width at half maximum of 0.5 eV.

3. For each excitation center i, an additional full core-hole calculation is done by using a pre-generated PAW potential. Further, an additional electron is added at the Fermi level. The resulting electronic energy, E^i_{ce} is used to calculate the first core excitation energy:

$$\Delta_i = E^i_{ce} - E_{gs} \quad . \tag{S8}$$

The spectrum is then shifted such that the first peak coincides with Δ_i.

4. To obtain the total X-ray absorption cross section, we sum over all individual contributions, i.e., all excitation centers:

$$\sigma_T(\omega) = \sum_i \sigma^i(\omega) \tag{S9}$$

The atom-resolved spectra are obtained by plotting each individual contribution $\sigma^i(\omega, t_n)$ together with the total spectrum $\sigma_T(\omega, t_n)$.

Finally, the time-averaged total spectra shown in Figs. 2(a)-(c) as black dashed lines are

calculated as:

$$\langle \sigma_T(\omega) \rangle = \sum_i \langle \sigma^i(\omega) \rangle\,, \tag{S10}$$

where $\langle \sigma^i(\omega) \rangle$ is the time-averaged contribution of atom i (colored curves in Figs. 2(a)-(c)), i.e.,

$$\langle \sigma^i(\omega) \rangle = \frac{1}{N} \sum_n \sigma^i(\omega, t_n)\ , \tag{S11}$$

with N the number of time steps included in the n-summation, i.e., in the time interval of interest.

Acknowledgement

A.T, J.I.J, and M.A. acknowledge financial support by the Gobierno Vasco-UPV/EHU [Project No. IT1569-22] and by the Spanish MCIN/AEI/10.13039/501100011033 [Grant No. PID2019-107396GB-I00]. P.S. acknowledges support by the Deutsche Forschungsgemeinschaft (DFG), through project Sa 547-18. C.E. acknowledges the Klaus Tschira Foundation for financial support. This research was conducted in the scope of the Transnational Common Laboratory (LTC) "QuantumChemPhys – Theoretical Chemistry and Physics at the Quantum Scale". Computational resources were provided by the DIPC computing center.

References

(S1) Alducin, M.; Camillone, N.; Hong, S.-Y.; Juaristi, J. I. Electrons and Phonons Cooperate in the Laser-Induced Desorption of CO from Pd(111). *Phys. Rev. Lett.* **2019**, *123*, 246802.

(S2) Anisimov, S. I.; Kapeliovich, B. L.; Perel'man, T. L. Electron Emission from Metal Surfaces Exposed to Ultrashort Laser Pulses. *Sov. Phys.-JETP* **1974**, *39*, 375.

(S3) Juaristi, J. I.; Alducin, M.; Díez Muiño, R.; Busnengo, H. F.; Salin, A. Role of Electron-Hole Pair Excitations in the Dissociative Adsorption of Diatomic Molecules on Metal Surfaces. *Phys. Rev. Lett.* **2008**, *100*, 116102.

(S4) Alducin, M.; Díez Muiño, R.; Juaristi, J. I. Non-adiabatic Effects in Elementary Reaction Processes at Metal Surfaces. *Prog. Surf. Sci.* **2017**, *92*, 317 – 340.

(S5) Zaremba, E.; Rose, J. H.; Sander, L. M.; Shore, H. B. Self-Consistent Screening of a Proton in Jellium. *J. Phys. F: Met. Phys.* **1977**, *7*, 1763.

(S6) Echenique, P. M.; Nieminen, R. M.; Ritchie, R. H. Density Functional Calculation of Stopping Power of an Electron Gas for Slow Ions. *Solid State Commun.* **1981**, *37*, 779 – 781.

(S7) Novko, D.; Blanco-Rey, M.; Juaristi, J. I.; Alducin, M. *Ab Initio* Molecular Dynamics with Simultaneous Electron and Phonon Excitations: Application To The Relaxation of Hot Atoms and Molecules On Metal Surfaces. *Phys. Rev. B.* **2015**, *92*, 201411.

(S8) Novko, D.; Blanco-Rey, M.; Alducin, M.; Juaristi, J. I. Surface Electron Density Models For Accurate *Ab Initio* Molecular Dynamics with Electronic Friction. *Phys. Rev. B.* **2016**, *93*, 245435.

(S9) Hirshfeld, F. L. Bonded-Atom Fragments For Describing Molecular Charge Densities. *Theoret. Chim. Acta* **1977**, *44*, 129.

(S10) Nosé, S. A Unified Formulation of the Constant Temperature Molecular Dynamics Methods. *J. Chem. Phys.* **1984**, *81*, 511–519.

(S11) Hoover, W. G. Canonical Dynamics: Equilibrium Phase-space Distributions. *Phys. Rev. A* **1985**, *31*, 1695–1697.

(S12) Hünenberger, P. H. In *Advanced Computer Simulation: Approaches for Soft Matter Sciences I*; Holm, C., Kremer, K., Eds.; Springer Berlin Heidelberg, 2005; pp 105–149.

(S13) Kresse, G.; Furthmüller, J. Efficiency of Ab-Initio Total Energy Calculations For Metals and Semiconductors Using a Plane-Wave Basis Set. *Comput. Mater. Sci.* **1996**, *6*, 15 – 50.

(S14) Kresse, G.; Furthmüller, J. Efficient Iterative Schemes For Ab Initio Total-Energy Calculations Using a Plane-Wave Basis Set. *Phys. Rev. B.* **1996**, *54*, 11169–11186.

(S15) Blanco-Rey, M.; Juaristi, J. I.; Díez Muiño, R.; Busnengo, H. F.; Kroes, G. J.; Alducin, M. Electronic Friction Dominates Hydrogen Hot-Atom Relaxation on Pd(100). *Phys. Rev. Lett.* **2014**, *112*, 103203.

(S16) Saalfrank, P.; Juaristi, J. I.; Alducin, M.; Blanco-Rey, M.; Díez Muiño, R. Vibrational Lifetimes of Hydrogen on Lead Films: An Ab Initio Molecular Dynamics with Electronic Friction (AIMDEF) Study. *J. Chem. Phys.* **2014**, *141*, 234702.

(S17) Novko, D.; Blanco-Rey, M.; Juaristi, J. I.; Alducin, M. Energy Loss in Gas-Surface Dynamics: Electron-Hole Pair and Phonon Excitation Upon Adsorbate Relaxation. *Nucl. Instrum. Methods B* **2016**, *382*, 26–31.

(S18) Novko, D.; Lončarić, I.; Blanco-Rey, M.; Juaristi, J. I.; Alducin, M. Energy Loss and Surface Temperature Effects in Ab Initio Molecular Dynamics Simulations: N Adsorption on Ag(111) as a Case Study. *Phys. Rev. B* **2017**, *96*, 085437.

(S19) Juaristi, J. I.; Alducin, M.; Saalfrank, P. Femtosecond Laser Induced Desorption of H_2, D_2, and HD from Ru(0001): Dynamical Promotion and Suppression Studied with Ab Initio Molecular Dynamics with Electronic Friction. *Phys. Rev. B* **2017**, *95*, 125439.

(S20) Dion, M.; Rydberg, H.; Schröder, E.; Langreth, D. C.; Lundqvist, B. I. Van der Waals Density Functional for General Geometries. *Phys. Rev. Lett.* **2004**, *92*, 246401.

(S21) Tetenoire, A.; Juaristi, J. I.; Alducin, M. Insights into the Coadsorption and Reactivity of O and CO on Ru(0001) and Their Coverage Dependence. *J. Phys. Chem. C* **2021**, *125*, 12614–12627.

(S22) Monkhorst, H. J.; Pack, J. D. Special Points for Brillouin-zone Integrations. *Phys. Rev. B* **1976**, *13*, 5188–5192.

(S23) Methfessel, M.; Paxton, A. T. High-precision Sampling for Brillouin-zone Integration in Metals. *Phys. Rev. B* **1989**, *40*, 3616–3621.

(S24) Blöchl, P. E. Projector Augmented-wave Method. *Phys. Rev. B* **1994**, *50*, 17953–17979.

(S25) Kresse, G.; Joubert, D. From Ultrasoft Pseudopotentials to the Projector Augmented-wave Method. *Phys. Rev. B* **1999**, *59*, 1758–1775.

(S26) Anders Nilsson, L. G. P.; Norskov, J. K. In *Chemical Bonding at Surfaces and Interfaces*; Elsevier,, Ed.; 2008.

(S27) Michele Aresta, E. Q., Angela Dibenedetto In *Reaction Mechanisms in Carbon Dioxide Conversion*; Springer,, Ed.; 2016.

(S28) Enkovaara, J.; Rostgaard, C.; Mortensen, J. J.; Chen, J.; Dułak, M.; Ferrighi, L.; Gavnholt, J.; Glinsvad, C.; Haikola, V.; Hansen, H. A. et al. Electronic Structure Calculations with GPAW: A Real-Space Implementation of the Projector Augmented-Wave Method. *J. Phys.: Condens. Mat.* **2010**, *22*, 253202.

(S29) Mortensen, J. J.; Hansen, L. B.; Jacobsen, K. W. Real-Space Grid Implementation of the Projector Augmented Wave Method. *Phys. Rev. B* **2005**, *71*, 035109.

(S30) Perdew, J. P.; Burke, K.; Ernzerhof, M. Generalized Gradient Approximation Made Simple. *Phys. Rev. Lett.* **1996**, *77*, 3865–3868.

(S31) Perdew, J. P.; Burke, K.; Ernzerhof, M. Generalized Gradient Approximation Made Simple [Phys. Rev. Lett. 77, 3865 (1996)]. *Phys. Rev. Lett.* **1997**, *78*, 1396–1396.

(S32) Ehlert, C.; Klamroth, T. PSIXAS: A Psi4 Plugin for Efficient Simulations of X-ray Absorption Spectra Based on the Transition-Potential and Δ-Kohn–Sham Method. *J. Comput. Chem.* **2020**, *41*, 1781–1789.
